# The Role of Nrf2 in Cardiovascular Function and Disease

**DOI:** 10.1155/2017/9237263

**Published:** 2017-09-14

**Authors:** Sandro Satta, Ayman M. Mahmoud, Fiona L. Wilkinson, M. Yvonne Alexander, Stephen J. White

**Affiliations:** ^1^Vascular Pathology Group, Centre for Biomedicine, School of Healthcare Science, Manchester Metropolitan University, Manchester M1 5GD, UK; ^2^Physiology Division, Zoology Department, Faculty of Science, Beni-Suef University, Beni-Suef 62514, Egypt

## Abstract

Free radicals, reactive oxygen/nitrogen species (ROS/RNS), hydrogen sulphide, and hydrogen peroxide play an important role in both intracellular and intercellular signaling; however, their production and quenching need to be closely regulated to prevent cellular damage. An imbalance, due to exogenous sources of free radicals and chronic upregulation of endogenous production, contributes to many pathological conditions including cardiovascular disease and also more general processes involved in aging. Nuclear factor erythroid 2-like 2 (NFE2L2; commonly known as Nrf2) is a transcription factor that plays a major role in the dynamic regulation of a network of antioxidant and cytoprotective genes, through binding to and activating expression of promoters containing the antioxidant response element (ARE). Nrf2 activity is regulated by many mechanisms, suggesting that tight control is necessary for normal cell function and both hypoactivation and hyperactivation of Nrf2 are indicated in playing a role in different aspects of cardiovascular disease. Targeted activation of Nrf2 or downstream genes may prove to be a useful avenue in developing therapeutics to reduce the impact of cardiovascular disease. We will review the current status of Nrf2 and related signaling in cardiovascular disease and its relevance to current and potential treatment strategies.

## 1. Introduction

The vascular endothelium modulates vascular structure, thrombolysis, vasoconstriction, and vasodilation and maintains internal homeostasis through synthesizing and releasing several active biomolecules [1]. A loss of function of the endothelium represents a key risk factor for cardiovascular disease (CVD) and initiates the development of atherosclerosis [2]. Endothelial dysfunction is associated with functional changes that diminish nitric oxide (NO) bioavailability and consequently leads to CVD [1]. Sustained failure to counteract the excessive production of reactive oxygen species (ROS) and dysregulation of the antioxidant defence system in the endothelium elicits cellular damage and dysfunction [2]. However, the original concept that all free radicals are damaging disease-causing entities have, over recent years, been replaced by an understanding of the important signaling role they play within and between cells. The production and control of free radicals need to be tightly regulated to prevent cytotoxicity, and the imbalance, caused by exogenous sources of free radicals with chronic upregulation and endogenous production, contributes to many pathological conditions and also to more general processes involved in aging [3–5]. There are multiple cellular defence strategies to prevent free radical toxicity, which are dynamically regulated to protect from oxidative insults and preserve cell function [6]. Nuclear factor erythroid 2-like 2 (NFE2L2; commonly known as Nrf2 [7]) has been identified as a major regulator of the oxidant/antioxidant balance.

The Nrf2 was first discovered in 1994 by Moi et al. during studies on regulation of the *β*-globin gene [7]. It was subsequently identified to be profoundly involved in the regulation of oxidant and antioxidant gene expression, through binding to the antioxidant response element (ARE) [8, 9]. Nrf2/ARE signaling is highly conserved in all species and controls a wide panel of genes that include cytoprotective and detoxifying phase II enzymes [10]. Nrf2 coordinates the cellular response to oxidative insults, preventing damage to cellular components sensitive to redox changes (i.e., proteins, lipids, and DNA).

## 2. Regulation of Nrf2 Activity

**Figure 1:**
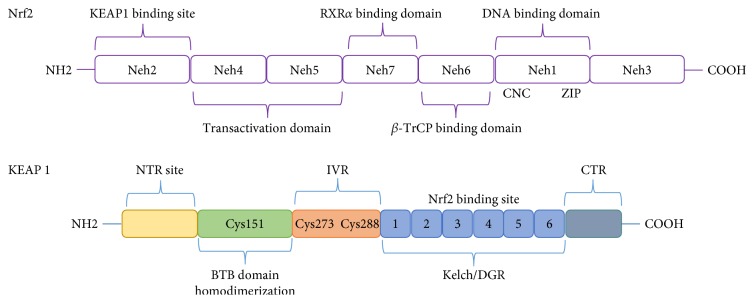
Nrf2 and KEAP1 structure. Nrf2 is a cap‘n'collar-basic region leucine zipper (CNC-bZIP), and its human sequence contains 605 amino acids, divided into seven domains: Neh1 to Neh7. Neh1 contains a CNC-bZIP motif, allowing heterodimerization with Maf proteins and DNA binding [54]. The Neh2 domain contains the Keap1 binding site (DLG and ETGE motifs), necessary for its cytoplasmic retention and degradation [55]. The Neh3 domain is fundamental for Nrf2 transcriptional activation through binding with chromo-ATPase/helicase DNA-binding protein 6 (CHD6) [56]. Neh4 and Neh5 provide an interaction site for nuclear cofactor RAC3/AIB1/SRC-3 [57] and CREB-binding protein (CBP) [58] which enhances the Nrf2/ARE activation pathways, partially by promoting acetylation of Nrf2 [59]. Additionally, Nrf2 possesses a redox-sensitive nuclear exporting signal within the Neh5 transactivation domain able to regulate its cellular localization [60]. The serine-rich Neh6 domain contains two motifs (DSGIS and DSAPGS) involved in the negative regulation of Nrf2. Glycogen synthase kinase 3 (GSK-3) phosphorylates serine residues within Neh6 enabling the interaction with the *β*-transducin repeat-containing protein (*β*-TrCP) which acts as a substrate receptor for Skp1–Cul1–Rbx1/Roc1 ubiquitin ligase complex, leading to KEAP1-independent degradation [41]. Neh7 domain interacts with retinoid X receptor alpha (RXR*α*), responsible for Nrf2/ARE signaling inhibition [61]. Human Kelch-like ECH-associated protein 1 (KEAP1) is a 69 kD protein, containing 27 cysteine residues. It is a substrate adaptor for cullin (Cul3) which contains E3 ubiquitin ligase (E3). KEAP1 is composed of five domains starting from the N-terminal region, a BTB dimerization domain (Broad-Complex, Tramtrack, and Bric-à-brac) which contains the Cys151 residue, a cysteine-rich intervening region (IVR) domain with two cysteine domain residues Cys273 and Cys288, critical for stress sensing. A Kelch domain/double glycine repeat (DGR) domain possessing 6 Kelch repeats and ending with a C-terminal region [62]. KEAP1 needs a domain capable to homodimerize and interact with Cul3, forming the Nrf2 inhibitor complex (iNrf2), and this is the BTB domain [63]. The Cys151 in the same domain plays an important role on Nrf2 activation in response to oxidative stress [64]. Furthermore, the IVR domain is highly sensitive to oxidation and contains three cysteines, 273, 288, and 297 which regulate Nrf2 activation and repression [16, 65]. The DGR domain acts as an Nrf2 repressor; it contains six repetitive Kelch structures that specifically bind to the Neh2 domain on Nrf2 [15].

Nrf2 activity is highly regulated, suggesting that either hypoactivation or hyperactivation of Nrf2 may be detrimental to the cell, for example, unrestricted Nrf2 activity, elicited by knockout of Kelch-like ECH-associated protein 1 (KEAP1) in the mouse, results in postnatal lethality [11], while Nrf2 knockouts are viable but hypersensitive to oxidative stressors. The regulation of Nrf2 has been extensively reviewed elsewhere [12–14] but is briefly summarized here and in Figure 1() and Table 1. *Nrf2-*regulated gene expression is primarily controlled by KEAP1. In a situation without oxidative stimuli, Nrf2 is mostly sequestered in the cytosol through binding to the Kelch domain of KEAP1 [15]. KEAP1 acts as an adapter molecule for CUL-E3 ligase and mediates the ubiquitination and degradation of Nrf2 protein. Exposure of oxidative/electrophilic stress causes a modification of the cysteine groups on KEAP1 (particularly C151), relaxing the structure of KEAP1 causing dissociation of KEAP1 from CUL-E3 ligase [16–18]. It is unclear if Nrf2 protein dissociates from KEAP1 or if modification of C151 simply blocks further processing of Nrf2 [18]. De novo synthesized Nrf2, or protein released from KEAP1, is then free to translocate to the nucleus. In addition, p21, p62, and the tumor suppressor WTX also potentiate Nrf2 activation through sequestration of KEAP1 or binding to Nrf2 to prevent association with KEAP1 [19–21]. Upon entry into the nucleus, Nrf2 heterodimerizes with a number of transcription factors, including small Maf proteins (allowing formation of full basic zipper, summarized in Figure 1() and Table 1), and binds to the ARE (core sequence RTGACnnnGCA) to induce gene transcription [22, 23].

Dissociation of KEAP1 from the CUL-E3 ligase complex can be induced by a large range of compounds, including oxidized phospholipids [24], nitric oxide (NO), zinc, alkenals [25], and cigarette smoke, or fresh aqueous extracts of cigarette smoke [26–28]. However, not all forms of ROS appear to be able to modify KEAP-1/Nrf2 interactions, with data suggesting this is both cell type and context specific. Of particular relevance to CVD, laminar shear stress causes the activation of Nrf2 in endothelial cells [29], through lipid peroxide and COX2-derived 15-deoxy-12,14-prostaglandin J2 (15d-PGJ2) intermediates, enhanced by phosphoinositol 3-kinase/Akt signaling, but is surprisingly independent of endothelial nitric oxide synthase (eNOS) activity [30–33]. In addition, laminar shear stress increases the nuclear localization of Nrf2 via a KLF2-dependent mechanism [34]. Finally, tumor necrosis factor alpha (TNF-*α*) increases the activation of Nrf2 in human endothelial cells [28] and monocytes [35]. A number of naturally occurring compounds have been shown to release Nrf2 from KEAP1 [36], for example, sulforaphane [37], sulfuretin [38], 2-trifluoromethyl-2-methoxychalone [39], and isoliquiritigenin [40], suggesting that dietary modulation of ARE-dependent gene expression could play a potential role in modulating disease.

## 3. Additional Regulatory Systems

In addition to KEAP1-mediated sequestration and degradation of Nrf2 within the cytoplasm, there are a number of additional layers of regulation on Nrf2-dependent gene expression. Degradation of Nrf2 can also be induced by *β*-TrCP–Skp1–Cul1–Rbx1 E3 ubiquitin ligase complex [41, 42], triggered by phosphorylation of Nrf2 within the Neh2 domain. Subsequently, the E3 ligase complex ubiquitinates Nrf2 and causes its destruction by the proteasome. Mitra et al. also observed that the inhibition of P38 mitogen-activated protein kinase (MAPK) highly decreased Nrf2 nuclear translocation, with a corresponding reduction of Nrf2-dependent gene expression [43]. While the majority of KEAP1 is normally present in the cytoplasm, 10–15% has localized to the nucleus [44]; prothymosin-alpha (ProT*α*) binds KEAP1, shuttling it into the nucleus, where it can bind Nrf2 and promote its degradation [45]. Within the nucleus, B-zip proteins BACH1 and BACH2 can form dimers with Maf proteins through their BTB domain and compete for binding to the ARE, preventing Nrf2 binding and activation of transcription [46–48]. BACH1 is universally expressed, while BACH2 expression is predominantly limited to monocytes and in neural cells. Phosphorylation of BACH1 on Y486 provokes nuclear export of BACH1 increasing Nrf2-dependent gene expression [49, 50]. Nuclear export of Nrf2 is controlled through a GSK-3*β*-controlled phosphorylation cascade. GSK-3*β* phosphorylates Src family kinases (Src, YES, and Fyn), in turn phosphorylating Nrf2 on Y568 triggering nuclear export and degradation [51–53].

## 4. Nrf2 and Mitochondrial Dynamics in Cardiovascular Disease

Cardiovascular disease is the main cause of death worldwide [78], and it covers a wide array of disorders. The most common causes of CVD morbidity and mortality are stroke, ischemic heart disease (IHD), and congestive heart failure (CHF). Several risk profiles are involved in CVD where ROS is a central mediator and a common denominator, upregulated by multiple risk factors such as diabetes, inflammation, and smoking [79–81]. ROS can cause EC apoptosis and activate nuclear factor kappa-B (NF-*κ*B), increasing adhesion molecules and cytokines that enhance monocyte adhesion [82, 83]. Oxidative stress is involved in mitochondrial dysfunction, which is related to bioenergetic defects and an alteration in mitochondrial dynamics. This provokes transcription impairment and cell damage. Blockage of the mitochondrial electron transfer in complex III in diabetes leads to the release of electrons which reduce molecular oxygen to superoxide (O_2_•) and increases intracellular ROS production [84]. Furthermore, ROS can activate membrane oxidases with a subsequent increase in the levels of asymmetric dimethylarginine that competes for the L-arginine transporters and active sites on eNOS [85]. Nrf2 modulates the activity of the mitochondrial respiration chain [86], with pharmacological activation of Nrf2 protecting against toxicity and maintaining mitochondrial homeostasis possibly via ablation of Akt2 signaling [87]. Liu and colleagues discovered acrolein, a component of cigarette combustion, inactivated the KEAP1/Nrf2 pathway, and decreased mitochondrial membrane potential [88], while Zou et al. demonstrated the ability of Nrf2 to prevent mitochondrial dysfunction, using hydroxytyrosol to activate Nrf2 [89].

## 5. Nrf2 in Endothelial Dysfunction

The vascular endothelium modulates vascular homeostasis through synthesizing and releasing several active biomolecules [1]. A loss of endothelium integrity represents a key risk factor for CVD, initiating atherosclerosis [2] and is associated with functional changes that diminish NO bioavailability and, consequently, lead to CVD [1]. Hypoxia, flow disturbances, and oxidative stress are important contributors to endothelial dysfunction [90]. Failure to counteract excessive production of ROS and modulation of the anti-oxidant defence system in the endothelium elicits cellular damage and dysfunction [2].

**Figure 2:**
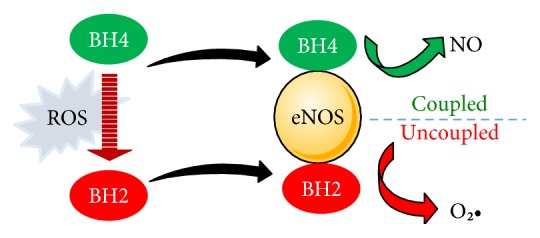
ROS-induced uncoupling of eNOS and the generation of O_2_•. Excess ROS induce the conversion of BH4 to BH2 with subsequent eNOS uncoupling and synthesis of O_2_• instead of NO. eNOS: endothelial nitric oxide synthase; ROS: reactive oxygen species; NO: nitric oxide; O_2_•: superoxide; BH4: tetrahydrobiopterin; BH2: dihydrobiopterin.

Normal vascular endothelial physiology is dependent on NO production via coupling of the eNOS heme group with L-arginine using tetrahydrobiopterin (BH4) as a cofactor [91]. Excess ROS induce the conversion of BH4 to 7,8-dihydrobiopterin (BH2) with subsequent eNOS uncoupling and synthesis of O_2_• instead of NO [91] (Figure 2()). O_2_• can react with NO to produce the versatile oxidant peroxynitrite (ONOO−) [92]. The upregulation of iNOS and uncoupling of eNOS under hyperglycemic conditions are now well established [93, 94]. L-arginine is also a substrate for arginase [95] which is upregulated in the endothelium of coronary arterioles in hypertension and contributes to the impaired NO-mediated dilation [96]. In addition, ONOO− and hydrogen peroxide (H_2_O_2_) were reported to increase the activity/expression of arginase in endothelial cells [97], thus exacerbating the defects in myogenic tone. Therefore, ROS can trigger eNOS uncoupling through depletion of the substrate L-arginine. This notion has been supported by the study of Romero et al. [98] where increased arginase activity elicited L-arginine depletion and contributed to endothelial dysfunction in diabetes. ONOO− can also activate NADPH oxidases and influences further generation of ROS [99]. Additionally, blockage of the mitochondrial electron transfer in complex III in diabetes leads to the release of electrons, which reduce molecular oxygen to O_2_• and increase intracellular ROS production [84]. Furthermore, ROS can activate membrane oxidases with a subsequent increase in the levels of asymmetric dimethylarginine that competes for the L-arginine transporters and active sites on eNOS [85].

Nrf2 in the endothelium can be activated via increased ROS production and PI3K-Akt signaling triggered by laminar shear stress [32]. In human arterial endothelial cells, Nrf2 activation resulted in increased intracellular HMOX1, GPx, GSH, GCLM, SRXN1, NQO1, PAR4, and OSGIN1 [27, 28, 100]. Adenoviral overexpression of Nrf2 in endothelial cells infected showed decreased expression of TNF-*α*, IL-1*β*, MCP1, and VCAM1, pointing to the anti-inflammatory potential of Nrf2 [28, 101]. When shear stress is disturbed at bifurcations, curved sections of arteries or distal to regions of stenosis, NO bioavailability decreases, O_2_• generation increases [102], and Nrf2-activated genes are diminished, causing the endothelium to become predisposed to atherogenesis [103]. Our recent studies have demonstrated that free fatty acid- (FFA-) induced excessive ROS production diminished both the gene and protein expression of Nrf2, NQO1, and HO-1 in endothelial cells [104]. In addition, upregulation of Nrf2/ARE/HMOX1 signaling protected the human endothelial cells against TNF-*α* activation [105]. It could be that mitochondrial ROS may trigger a protective response via Nrf2 activation in endothelial cells. The study of Lo and Hannink [106] suggested that Nrf2–KEAP1 complex binds to the mitochondria through interaction with mitochondrial outer membrane protein PGAM5 and directly senses mitochondrial ROS production.

Another possibility through which Nrf2 can protect the endothelium against the cytotoxic ROS involves regulating the catalytic subunit of GCLC which reduces GSH biosynthesis [107]. In this context, impaired Nrf2–KEAP1–GCLC has been demonstrated in high glucose-induced retinal endothelial cells from diabetic donors [108]. In the human brain microvascular endothelial cells (HBVEC), GSH conferred protection against FFA-induced oxidative stress and apoptosis by activating the Akt pathway [109]. Human umbilical vein endothelial cells (HUVECs), human coronary artery endothelial cells (HCAECs), and endothelial progenitor cells exposed to cytotoxic ROS showed apoptosis and cell death accompanied by diminished nuclear localization and transcriptional activity of Nrf2 [2]. These findings highlight the crucial role of Nrf2 activation in protecting endothelial cells against oxidative stress-induced dysfunction.

## 6. Nrf2 in Atherosclerosis

Atherosclerosis is a focal inflammatory disease of the arterial system involving a number of different cell types. The focal nature of atherosclerosis highlights the involvement of local haemodynamics factors acting on the endothelium in the initiation and progression of atherosclerosis, which develops in regions that experience disturbed flow at bifurcations and curved sections of artery [110–113]. Straight sections of artery that experience normal laminar blood flow are relatively spared from disease through a coordinated modulation of gene expression, predominantly controlled by the transcription factors KLF2 and KLF4 and activation of Nrf2 [29, 32, 114–116]. By contrast, endothelial cells exposed to disturbed flow adopt a phenotype that amplifies endothelial dysfunction and increases permeability. While ROS are essential signaling molecules regulating vascular homeostasis, excessive ROS, elevated by many of the risk factors associated with the development of atherosclerosis, promote endothelial dysfunction and decrease NO availability. Thus, Nrf2-regulated antioxidant gene expression may play an atheroprotective role in endothelial cells.

Consistent with this hypothesis, the Nrf2-regulated gene, heme oxygenase 1 (HMOX1), demonstrates significant cytoprotective and anti-inflammatory effects that result in a reduction of atherosclerosis in mouse models [117], possibly through production of low levels of carbon monoxide. Hypercholesterolemic mice, deficient in both HMOX1 and ApoE (HMOX1^−/−^/ApoE^−/−^), demonstrated enhanced development of atherosclerosis compared to ApoE^−/−^ single knockout mice [118]. HMOX1 expression in macrophages plays a protective role in atherosclerosis [119] with macrophages from HMOX1^−/−^ mice displaying increased ROS generation, production of inflammatory cytokines, and increased foam cell formation when treated with oxLDL, attributable in part to increased expression of scavenger receptor A (SR-A). Smooth muscle cells from HMOX1^−/−^ mice not only displayed increase neointimal formation but also enhanced cell death potentially due to greater susceptibility to oxidant stress [118]. Pharmacological modulation of HMOX1 expression also demonstrates a protective role of HMOX1 in atherogenesis [120, 121]. In addition to the anti-inflammatory effects of carbon monoxide, hydrogen sulphide also elicits an anti-inflammatory antiatherogenic effect [122]. Hydrogen sulfide activates the release of Nrf2 from KEAP1, increasing Nrf2-dependent gene expression [122].

Despite the antioxidant function of Nrf2 and the antiatherogenic function of the key Nrf2 target gene HMOX1, the global knockout of Nrf2 (Nrf2^−/−^) developed less rather than more atherosclerosis [123, 124]. Barajas et al. attributed this to an effect of Nrf2 in lipid metabolism, lowering plasma cholesterol and reducing foam cell formation [123], while Sussan et al. did not find a difference in serum cholesterol but attributed the effect to a reduction in scavenger receptor CD36 reducing foam cell formation [124]. The role of Nrf2 in NLRP3 inflammasome induction by cholesterol crystals within the atherosclerotic plaque may also be a contributing factor that explains the counterintuitive net detrimental effect of Nrf2 in hypercholesterolemic mouse models of atherosclerosis [125]. It might also explain why the expression of the Nrf2-regulated gene HMOX1 is highest in human plaques with the highest markers of plaque instability [121].

## 7. Nrf2 in Vascular Calcification

The presence of vascular calcification is often detected in atherosclerotic plaques and in patients with end-stage renal disease. Both of these pathologies have been targeted for prevention using pharmacological and genetic approaches by modulation of Nrf2 antioxidant pathways. For example, studies *in vitro* using rodent vascular smooth muscle cells show that dimethyfumarate or resveratrol could attenuate the deposition of a mineralised matrix and suggest protection against oxidative stress-induced mitochondrial damage, via activation of Nrf2 and SIRT1 signaling and downregulation of osteogenic transcription factors [126, 127]. In contrast, glucose-induced oxidative stress enhances the osteogenic differentiation and mineralisation of human embryonic stem (ES) cells, by the upregulation of runt-related transcription factor 2 (Runx2), Nrf2, and HMOX1, which was inhibited by Nrf2 knockdown [128] highlighting a context-specific regulation of the calcification process. Given the links between Nrf2 and bone homeostasis, it is not surprising to have an association between Nrf2 signaling and vascular calcification. Whether these initial *in vitro* studies can translate into the *in vivo* situation needs further study.

## 8. Nrf2 in Hypertension

Angiotensin II and associated renin-angiotensin system (RAS) are involved in the regulation of blood pressure, vasoconstriction, sodium intake, and potassium excretion [129]. Inappropriate activation of the RAS is the main cause of profound hypertension and cardiovascular morbidity. Angiotensin II increases the expression of NADPH oxidase and the generation of ROS potentially mediating some of the effects in renin-angiotensin-induced hypertension [130, 131]. It has been suggested that hypertension could be one of the causes of Nrf2 misregulation and not vice versa [132] through enhanced oxidative stress and vascular dysfunction in a hypertensive rat model [133]. This would suggest that the Nrf2 anti-oxidant defence system is insufficient to counteract the effects of oxidative stress, possibly due to elevated levels of Nrf2 repressors in hypertensive animals. Research is now moving from the adaptive and protective changes in the Nrf2 antioxidant response to focusing on the alternative mechanisms intrinsic to upstream and downstream molecules associated with a defective Nrf2 signaling system. Enhancing Nrf2 activity may have a therapeutic potential for a meliorating hypertension.

## 9. Nrf2 in Diabetic Cardiomyopathy

**Figure 3:**
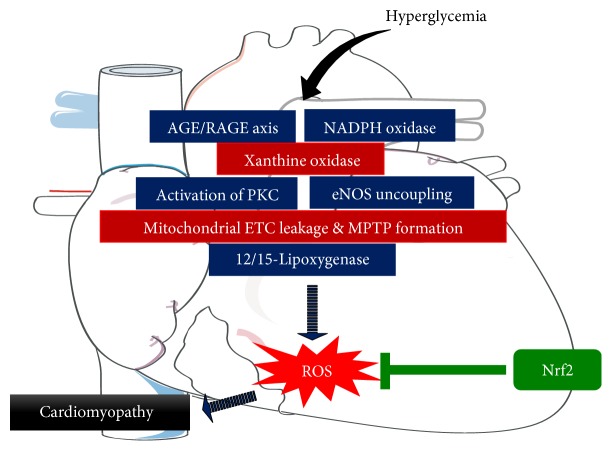
Hyperglycemia-induced ROS generation in the heart. A schematic model showing the potential pathways involved in cardiomyopathy and how Nrf2 could be targeted to reduce ROS and prevent the development of this pathology. AGEs: advanced glycation end products; NADPH: nicotinamide adenine dinucleotide phosphate; PKC: protein kinase C; eNOS: endothelial nitric oxide synthase; ETC: electron transport chain; MPTP: mitochondrial permeability transition pore.

The heart is particularly vulnerable to oxidative damage compared to other organs, due to its low basal levels of antioxidant defences [134]. Diabetic cardiomyopathy (DCM) and other cardiovascular complications account for more than 80% of deaths among patients with diabetes [135]. DCM is characterized by impaired diastolic function, hypertrophy, apoptosis, and fibrosis of cardiomyocytes [136] and involves several mechanisms and pathogenic factors, with oxidative stress thought to be the common link [137–140]. Hyperglycemia generates excess ROS/RNS from activation of NADPH oxidases, PKC, leakage of the mitochondrial electron transport chain, eNOS uncoupling, AGE/RAGE signaling, xanthine oxidase, and 12/15-lipoxygenase (LOX) [141], impairing antioxidant defences in the diabetic heart [138, 140] (Figure 3).

Studies have established the importance of Nrf2/ARE signaling in the prevention of diabetic complications [142–144] and oxidative stress-induced cardiomyocyte injury [145, 146]. Significantly reduced Nrf2 expression has been observed in the left ventricle of diabetic patient heart by histological analysis [147], which has also been observed in a diabetic mouse model after 5 months [147]. These findings suggest adaptive overexpression of Nrf2 to combat early oxidative damage in diabetes, which is overcome by sustained ROS production and exhaustion of the antioxidant defences [148]. This concept is supported by our findings in palmitate-treated endothelial cells, where reduced Nrf2 expression and antioxidant defences are observed with surplus ROS [104].

Furthermore, it has been demonstrated that Nrf2 and its downstream target genes are downregulated in cardiomyocytes from diabetic (*db/db*) mice [146, 147], which may occur via extracellular signal-regulated protein kinase (ERK) 1/2 activity [149, 150]. Isoproterenol-stimulated contraction of primary cardiomyocytes from adult diabetic mice was also shown to be dependent on Nrf2 [151]. Hence, hyperglycemia-induced loss of Nrf2 function exacerbates oxidative stress and leads to severe myocardial damage [151]. Nrf2 knockout mice exhibit structural and functional abnormalities under conditions of pathological stress [152], and cardiomyocytes from Nrf2-knockout mice showed significantly increased apoptosis following incubation with high glucose [151]. These findings highlight the importance of the Nrf2 protective mechanisms, and thus, novel therapeutics to enhance Nrf2 could be beneficial in this scenario. The proteasome inhibitor MG-132, which increases Nrf2 signaling, was reported to decrease left ventricle hypertrophy by reducing inflammation and lowering the risk of cardiomyopathy [153]. In addition, in a mouse model of type I diabetes mellitus, Nrf2 activation by sulforaphane reduced heart weight and decreased diabetes-induced atrial natriuretic peptide (ANP) expression, thought to be related to induction of DCM [154]. Therefore, enhancing endogenous Nrf2 and subsequent antioxidant pathways in the heart is a potential strategy to prevent DCM [138, 155].

## 10. Nrf2 in the Aging Heart

Aging, a progressive decline of cellular functions, is related to the loss of homeostasis via a combination of epigenetic alterations and genetically programmed processes resulting in death [156, 157]. Heart capacity declines with age, with a concomitant increased CVD risk [158]. Herman's free radical theory proposes that the accumulation of damaged biomolecules by ROS/RNS plays a central role in aging [159–161]. In turn, this leads to activation of NF-*κ*B [162], eliciting an inflammatory response via TNF-*α*, IL-6, and C reactive protein (CRP), reported to be associated with aging [163], and further stimulation of ROS production through activation of NADPH oxidase [164, 165] and NF-*κ*B [166]. In support of this, elderly patients demonstrate an impaired endothelial-dependent dilation, associated with excess ROS, activated NADPH oxidase, and increased NF-*κ*B [167].

Elevated ROS also increase the rate of apoptosis and necrosis in cardiomyocytes [168], resulting in functional and phenotypic changes, including decreased remodelling [169], cardiac hypertrophy [170], and increased systolic pressure [171, 172]. NADPH oxidase-2, its activator RAC1, and several profibrotic factors are elevated in hypertrophic hearts in aged rats [158], pointing to the important role of NADPH oxidase in aging-associated cardiomyocyte remodelling. Ischemia and reperfusion are characterized by increased accumulation of intracellular Ca^2+^, altered substrate utilization, and elevated ROS production in the heart [173], which can damage ionic pumps and induce mitochondrial dysfunction via lipid peroxidation [174]. This damage can lead to necrotic cell death [175] and is exacerbated with aging [160, 176], as shown in mitochondria from aged rats [177].

Diminished activity of Nrf2 resulting in oxidative stress, apoptosis, and/or necrosis in the myocardium has been reported [178–180], thus predisposing the heart to disease [180]. Studies in mouse models have supported the notion that Nrf2 is involved in counteracting aging-associated cardiac effects via ARE signaling and expression of antioxidant enzymes. Bailey-Downs et al. [181] reported increased sensitivity of blood vessels to stress-induced damage along with impaired activity of Nrf2 in insulin-like growth factor 1 (*Igf1*) knockout mice, promoting an aging phenotype. Nrf2-knockout mice showed exaggerated cardiac hypertrophy, heart failure, increased mortality [152], and oxidative stress [182]. Aged rhesus macaques have shown increased ROS and decreased nuclear translocation of Nrf2 and protein expression of NQO1 and HO-1 in their carotid arteries [183]. Vascular smooth muscle cells (VSMCs) derived from old monkeys have exhibited diminished Nrf2 activation following incubation with high glucose as compared with those derived from younger monkeys [183]. Additionally, El Assar et al. [165] have reported a decreased expression of Nrf2-regulated antioxidants in aged vessels.

These data demonstrate clearly that decreasing levels of Nrf2 are age-dependent but may be reversed by exercising. Muthusamy et al. [184] demonstrated an increased nuclear translocation of Nrf2 in the hearts of mice following acute exercise training. They attributed their findings to the induction of an exercise-induced mild oxidative state. Endurance exercise training was reported to promote Nrf2 signaling and enhance antioxidant capacity in the hearts of 6-month-old mice [185], which might offset the reduced signaling observed in aged mice and men [171, 185–187].

## 11. Role of Nrf2 Activation in the Treatment of CVD

The role of activators of Nrf2 in attenuating oxidative stress-mediated cardiovascular disorders has been identified. In Table 2, we present a summary of the recently studied activators of Nrf2 and their beneficial effects in CVD.

## 12. Conclusions

The Nrf2 antioxidant system plays a significant role in cellular defence against free radical damage, while insufficiency of Nrf2-dependent gene expression is clearly implicated in multiple aspects and stages of CVD. Enhancing Nrf2 activity may be beneficial in diabetic cardiomyopathy, mitochondrial dysfunction, and reducing the effects of aging in the heart; however, the potential exacerbation of atherosclerosis by Nrf2-mediated inflammasome activation in plaque macrophages, along with the lethality of KEAP1 knockout mice, raises a cautionary note to pharmacological activation of Nrf2 as a therapeutic strategy. Selective upregulation of Nrf2 target genes such as HMOX1 may provide a more amenable therapeutic strategy. Modest activation of Nrf2 by dietary factors, such as sulforaphane, found in brassicas like broccoli, may highlight mild activation of Nrf2 as part of the protective role played in eating a healthy balanced diet, which may be sufficient to maximise the therapeutic benefit offered through the control of this gene expression network.

## Figures and Tables

**Table 1:** List of proteins that bind to and modulate the activity of Nrf2.

Gene	Function	Reference
KEAP1	Retention in cytoplasm and degradation	[15]
CDH1/CTNNB1	Enhances KEAP1 interaction	[66]
CRF1	Ubiquitination and degradation	[67]
ATF4	Activation of gene expression	[68]
BRG1	Selective activation of gene expression	[69]
CBP	Activation of gene expression	[58]
CHD6	Activation of gene expression	[56]
JUN	Activation of gene expression	[9]
MAFF	Heterodimer activates gene expression	[70]
MAFG	Heterodimer activates gene expression	[71]
MAFK	Heterodimer activates gene expression	[71]
PMF1	Activation of gene expression	[72]
RAC3/AIB1/SRC-3	Activation of gene expression	[57]
PKC	Phosphorylation increases nuclear translocation	[73, 74]
HDAC1/2/3	Repression of gene expression	[75]
MYC	Repression of gene expression	[76]
PPARG	Repression of gene expression	[77]
RXR*α*	Repression of gene expression	[61]
FYN	Phosphorylation and nuclear export	[52]
SRC	Phosphorylation and nuclear export	[53]
YES	Phosphorylation and nuclear export	[53]

**Table 2:** The effect of Nrf2 activation on CVD.

Activator	Animal model/cell line	Effects	Reference
Bardoxolone methyl derivative dh404	Male Akita mice at 26 weeks of age & human aortic endothelial cells (HAECs)	Attenuation of endothelial dysfunction Downregulation of inflammatory and prooxidant genes Reduction in systemic and vascular oxidative stress	[188]
	Streptozotocin- (STZ-) induced diabetic ApoE^−/−^ mice	Prevention of atherosclerosis	[189]

Sulforaphane	Vascular smooth muscle cells (VSMCs)	Suppression of VSMC proliferation	[190]
	HUVECs	Protection against oxidized low-density lipoprotein- (oxLDL-) induced endothelial damage	[191]
	High-fat diet- (HFD-) induced type 2 diabetic mice	Prevention of aortic damage	[192]
	Low-dose STZ diabetic mice	Prevention of diabetic cardiomyopathy	[154]
	Multiple low dose STZ-induced type 1 diabetic mice	Prevents aortic oxidative damage, fibrosis, and inflammation	[193]

Miltirone	EA.hy926 endothelial cells	Protects against oxLDL-derived oxidative stress	[194]

Epigallocatechin-3-gallate	HUVECs	Protects against PM2.5-induced oxidative stress	[195]

*Barleria lupulina* alkyl catechols (4-ethylcatechol, 4-vinylcatechol, and 4-methylcatechol)	Human dermal microvascular endothelial cells	Improves organization of the cytoskeleton Organizes tight cell junctions Reduces inflammation and vascular leakage	[196]

Small molecule glycomimetics	HUVECs	Attenuates palmitate-induced oxidative stress and endothelial dysfunction. Increases NO production.	[104]

Rutin	HUVECs	Prevents hydrogen peroxide- (H_2_O_2_-) induced oxidative stress	[197]

1,25-Dihydroxycholecalciferol	HUVECs	Prevents leptin-induced oxidative stress and inflammation	[198]

Willow bark extract	HUVECs and *Caenorhabditis elegans*	Prevents ROS-induced cytotoxicity of HUVECs and death of *C. elegans*	[199]

Aged garlic extract	HUVECs	Enhances HO-1 and glutamate-cysteine ligase modifier subunit expression (GCLM)	[200]

Celastrol	HUVECs	Attenuates angiotensin II mediated endothelial damage	[201]

Paeotang	HUVECs	Prevents TNF-*α*-induced vascular inflammation	[202]

Cyanidin-3-O-glucoside	HUVECs	Ameliorates palmitate-induced insulin resistance and endothelial derived vasoactive factors	[203]
		Attenuates palmitate-induced inflammation	[204]
	EA.hy926 endothelial cells	Attenuates angiotensin II-induced oxidative stress and inflammation	[205]

Piceatannol	HUVECs	Attenuates homocysteine-induced endoplasmic reticulum stress and cell damage	[206]

Equol	ApoE^−/−^ mice	Attenuates atherosclerosis and inhibits endoplasmic reticulum stress	[207]
	HUVECs	Abrogates apoptosis induced by t-BHP	

Sheep/goat whey protein	EA.hy926 endothelial cells	Increases antioxidant defences	[208]

Quercetin	HAECs	Inhibits LPS-induced adhesion molecule expression and ROS production	[209]

Panax notoginseng saponins and Ginsenoside Rb1	HUVECs	Suppresses monocyte adhesion and inhibits ROS	[210]

Bortezomib	Human microvascular endothelial cells (HMECs)	Induces expression of HO-1	[211]

Sofalcone	HUVECs	Suppresses endothelial dysfunction	[212]

Salidroside	HUVECs	Suppresses ROS-induced damage	[213]

Caffeic acid	HUVECs	Attenuates high glucose-induced endothelial dysfunction	[214]

Myricitrin	H9c2 cardiomyocytes	Attenuates high glucose-induced apoptosis	[215]
	STZ-induced diabetic mice & AGE-induced H9c2 cardiomyocytes	Alleviates oxidative stress-induced inflammation, apoptosis, and cardiomyopathy	[216]

Andrographolide	EA.hy926 endothelial cells	Inhibits hypoxia-induced HIF-1*α*-driven endothelin 1 secretion	[217]

Tanshinone IIA	HUVECs	Inhibits cyclic strain-induced expression of interleukin 8	[218]

Lycopene	HUVECs	Inhibits cyclic strain-induced endothelin-1 expression and oxidative stress	[219]

Withaferin A	EA.hy926 endothelial cells & HUVECs	Induces HO-1 expression	[220]

Copper diethyldithiocarbamate	Bovine aortic endothelial cells	Inhibits proteasome and Nrf2 binding to Kelch-like ECH-associated protein 1	[221]

Clopidogrel	HAECs	Hinders TNF-*α*-induced VCAM-1 expression	[222]

*Hericium erinaceus*	EA.hy926 endothelial cells	Inhibits TNF-*α*-induced angiogenesis and ROS generation	[223]

Andrographolide	Primary cerebral endothelial cells	Prevents middle cerebral artery occlusion- (MCAO-) induced ischemic stroke	[224]

Butin	C57/BL6J diabetic mice	Prevents ischemia/reperfusion-induced myocardial injury	[225]

Aspalathin	H9c2 cardiomyocytes and diabetic db/db mice	Protects against hyperglycemia-induced oxidative damage and apoptosis	[146]

Broccoli sprout	Diabetic db/db mice	Prevents diabetic cardiomyopathy	[226]

Oleuropein	Spontaneously hypertensive rats	Attenuates oxidative stress and improves mitochondrial function in the hypothalamic paraventricular nucleus	[227]

Aralia taibaiensis	H9c2 cardiomyocytes	Protects against high glucose-induced oxidative stress and apoptosis	[228]

Compound C66	STZ-induced diabetic mice aorta	Prevents oxidative and nitrative stress, inflammation, apoptosis, cell proliferation, and fibrosis	[229]

Dimethyl fumarate	VSMCs	Attenuates vascular calcification	[127]

Gemigliptin	VSMCs	Prevents proliferation and migration of VSMCs	[230]

L6H9 (chalcone)	H9c2 cardiomyocytes	Prevents hyperglycemia-induced oxidative stress and inflammation	[231]

Magnesium lithospermate B	VSMCs	Prevents proliferation and migration of VSMCs	[232]

4-O-methylhonokiol	HFD-induced obese mice	Prevents cardiac pathogenesis	[233]

Resveratrol	Coronary arterial endothelial cells	Protects against high glucose-induced endothelial protection	[234]
	VSMCs	Ameliorates vascular calcification	[126]
	db/db mice	Ameliorates vascular inflammation	[235]
	STZ-induced type 2 diabetic rats	Ameliorates vascular inflammation	[236]

MG132	OVE26 type 1 diabetic mice	Prevents aortic oxidative damage and inflammatory response	[237]
		Prevents cardiomyopathy	[238]
